# Quality of life in children with infantile hemangioma: a case control study

**DOI:** 10.1186/s12955-017-0772-z

**Published:** 2017-11-16

**Authors:** Chuan Wang, Yanan Li, Bo Xiang, Fei Xiong, Kai Li, Kaiying Yang, Siyuan Chen, Yi Ji

**Affiliations:** 1 Division of Oncology, Department of Pediatric Surgery, West China Hospital of Sichuan University, #37 Guo-Xue-Xiang, Chengdu, Sichuan Province 610041 China; 2 Department of Child Health, West China Second University Hospital, Sichuan University, Sichuan Province, Chengdu, China; 3 Division of Oncology, Department of Pediatric Surgery, Children’s Hospital of Fudan University, Shanghai, China; 4 Pediatric Intensive Care Unit, Department of Critical Care Medicine, West China Hospital of Sichuan University, Chengdu, Sichuan Province China

**Keywords:** Infantile hemangioma, Quality of life, QOL instrument for IH, Pediatric quality of life inventory, Hemangioma location

## Abstract

**Background:**

Infantile hemangioma (IH) is the most common vascular tumor in children. It is controversial whether IHs has effects on the quality of life (QOL) in patients of whom IH poses no threat or potential for complication. Thus, we conducted this study to evaluate the q QOL in patients with IH and find the predictors of poor QOL.

**Methods:**

The PedsQL 4.0 Genetic Core Scales and the PedsQL family information form were administered to parents of children with IH and healthy children both younger than 2-year-old. The quality-of-life instrument for IH (IH-QOL) and the PedsQL 4.0 family impact module were administered to parents of children with IH. We compared the PedsQL 4.0 Genetic Core Scales (GCIS) scores of the two groups. Multiple step-wise regression analysis was used to determine factors that influenced QOL in children with IH and their parents.

**Results:**

Except for physical symptom, we found no significant difference in GCIS between patient group and healthy group (*P* = 0.409). The internal reliability of IH-QOL was excellent with the Cronbach’s alpha coefficient for summary scores being 0.76. Multiple step-wise regression analysis showed that the predictors of poor IH-QOL total scores were hemangioma size, location, and mother’s education level. The predictors of poor FIM total scores were hemangioma location and father’s education level. The predictors of poor GCIS total scores were children’s age, hemangioma location and father’s education level.

**Conclusion:**

The findings support the feasibility and reliability of the Chinese version of IH-QOL to evaluate the QOL in children with IH and their parents. Hemangioma size, location and education level of mother are important impact factors for QOL in children with IH and their parents.

## Background

Infantile hemangioma (IH) is the most common vascular tumor in children, with an estimated prevalence of 5 to 10%. IH usually presents at birth, develops rapidly in the first few months and involutes gradually at last. Depending on the appearance of IH and dictated by the depth, location, and stage of growth, the lesions are heterogeneous [1–3]. In most patients, the hemangioma lesions are small and pose no threat or potential for scarring and complication. However, in some cases, IHs can grow dramatically, destroy tissues and organs, impair function, or even threaten the patients’ lives [4]. Importantly, IHs are also able to cause physical distress and emotional concern to both children and their families, and all of these eventually influence quality of life (QOL) [5].

Improvement in QOL of patients and their families is one of the most significant outcomes of treatment in pediatric tumors. QOL of patients and their families is able to reflect the efficacy of treatment as well. Previously, investigators did not have validated disease-specific instrument to evaluate the QOL in IH patients and their families. As results, the authors could not directly ask the patients and/or their parents about the influence of IHs. Studies using the generic QOL instruments have yielded conflicting results [6, 7]. By using a Dutch version of hemangioma-specific questionnaires, Hoornweg et al. demonstrated that most patients’ QOL was not affected by IH [6]. However, the authors found considerable psychosocial impact of IH on patients, especially in those with complication or IH was visible located. Until recently, an English version of QOL instrument for IHs (IH-QOL) has been developed [8]. This instrument provides the required information account for the physical, emotional, social and cognitive development that takes place during the early critical period.

Until now, there is little information available on QOL in IH patients younger than 2 years old - when most of the IH growth has already occurred [9]. This is also the period that patients are most likely to undergo treatment, particularly in current ‘propranolol era’ [2]. It is still controversial whether IHs has effects on QOL in cases with small lesions. In addition, questions exist as to which factors are associated with QOL in IH patients and their parents.

The goal of the present study was to evaluate and analyze QOL in IH patients younger than 2 years old, with the aim to improve our understanding of the effects of IH on QOL in children and their parents.

## Methods

### Participants and procedures

This study was approved by the Ethics Committee of the West China Hospital of Sichuan University. All procedures followed the research protocols approved by West China Hospital of Sichuan University and West China Second University Hospital of Sichuan University and were conducted according to the Declaration of Helsinki. All patients were recruited from August 2015 and June 2016, at Department of Pediatric Surgery, West China Hospital of Sichuan University. The criteria for inclusion were as follows: 1) The patients were less than 2 years of age; 2) Patients were currently not received treatment; and 3) Consent of parents. The exclusion criteria were: 1) Patients were older than 2 year at the time of interview; 2) Patients had hemangioma-unrelated comorbid disease or major development disorders. Age and gender matched control subjects were healthy children aged 0 to 2 years. They were recruited from Department of Child Health, West China Second Hospital of Sichuan University, between August 2015 and June 2016.

Written informed consents were obtained from all children’s parents. The location and size of hemangioma were noted. In the case of multiple IHs, only the clinically most important IH (typically the largest or visceral involvement) was documented. The parents completed the questionnaires during the patients’ outpatient department visit. Questionnaires contained Chinese versions of IH-QOL and the Pediatric Quality of Life Inventory™ (PedsQL™). PedsQL™ consisted of the PedsQL™ Family Information Form (FIF), the pedsQL™ 4.0 Family Impact Module (FIM), the PedsQL™ 4.0 Generic Core Infant Scales (GCIS) 1–12 months and the PedsQL™ 4.0 Generic Core Infant Scales 13–24 months. The parents of children in patient group were required to complete the IH-QOL, the PedsQL™ FIF, the pedsQL™ FIM and the PedsQL™ 4.0 GCIS. The parents of children in healthy group were required to complete the PedsQL™ FIF and the PedsQL™ 4.0 GCIS. The investigators were trained by the project manager in order to guarantee the quality of the investigation. If the parents had questions on semantic or conceptual understanding, the investigators assisted parents to complete the questionnaires. Besides, the investigators were responsible to ensure there were no missing data in questionnaires.

### Instruments

The PedsQL™ 4.0 GCIS 1–12 months and the PedsQL™ 4.0 GCIS 13–24 months are brief, parent-proxy-reported standardized questionnaires. They are widely used to assess QOL in pediatric patients [10]. The module encompasses 5 functional domains: physical functioning, physical symptom, emotional functioning, social functioning and cognitive functioning. The PedsQL™ 4.0 GCIS 1–12 months contains 36 items and the PedsQL™ 4.0 GCIS 13–24 months contains 45 items. The Chinese version of PedsQL™ 4.0 GCIS has been demonstrated to have good internal consistency, as well as discriminant and construct validity for other disease [11, 12].

The PedsQL™ 4.0 FIF, which was cross-culturally adapted into Chinese, is aimed at collecting a child’s basic information, including date of birth, gender and disease duration, as well as caregiver’s basic information including marital status and education levels.

The PedsQL™ FIM, which was developed by Varni et al. [11–13], applies to measuring the impact of pediatric chronic disease on their family functioning. Chen et al. have translated it cross-culturally into Chinese version [11, 12, 14]. This instrument is composed of 9 dimensions (physical functioning, emotional functioning, social functioning, cognitive functioning, communication, worry, daily activities, family relationships and financial issues) and 37 items. The FIM can stand alone, or be integrated into the other measurement model, allowing an overall assessment of QOL of children and their parents.

The IH-QOL, which was developed by Sarah L Chamlin et al., is designed to measure the impact on IH patients and their parents [8]. This module consists of 4 domains (physical symptom of patient, social functioning of patient, social and psychological functioning of caregiver, and emotional functioning of caregiver) and 29 items. The Chinese Mandarin Version of IH-QOL was translated according to the standardized procedures and consisted of 4 steps: forward translation (Chinese), backward translation (English), preliminary test and field test.

Likert-type scale responses are used in all questionnaires: 0 = never a problem, 1 = almost never a problem, 2 = sometimes a problem, 3 = often a problem and 4 = almost always a problem. Scores of each item were linearly transformed into a 0 to 100 scale (0 = 100, 1 = 75, 2 = 50, 3 = 25, 4 = 0).

### Statistical analysis

All analyses were conducted using SPSS 22.0 for Windows (SPSS Inc., Chicago, USA). Chi-square (χ^2^) tests were used to compare qualitative variables. Independent sample *t*-test was used to analyze the continuous variables. Pearson correlation coefficients were used to evaluate the scaling success, with value ranging 0.6 to 0.8 presenting strong correlation, and ranging 0.4 to 0.6 presenting moderate correlation. Internal consistency reliability of IH-QOL was determined by calculating Cronbach’s alpha coefficient. For IH-QOL total score, Cronbach’s alpha coefficient ranging from 0.7 to 0.8 presented good reliability, and from 0.65 to 0.7 presented moderate reliability. For subscales, Cronbach’s alpha coefficient ranging from 0.7 to 0.8 presented good reliability, and from 0.6 to 0.7 presented moderate reliability. Multiple step-wise regression analyses were used to find out the main influencing factors for GCIS, FIM and IH-QOL total scores [15]. Statistical significance was set at *P* < 0.05.

## Results

Two hundred and seventeen children (76 male, 141 female) with IH were included. Ninety-five healthy children (39 male, 56 female) were selected as the healthy control group. Comparisons of demographic characteristics in two groups are listed in Table 1. There were no significant differences in demographic characteristics between patient group and healthy children (*P* > 0.05). The clinical characteristics and demographic details of patient group are listed in Table 2. The craniofacial and neck area (109) was the dominant location, representing 50% of all IHs, followed by the trunk (44), extremities (36), visceral involvement (16) and diaper area (12). Internal organ involvement in these children with IH contained 14 liver involvement, and 2 subglottie involvement. None of these 16 children developed complications.

**Table 1:** Demographics of patients and healthy children

Variables	IH patients	Healthy children	*P*-values
	*n* = 217	*n* = 95	
Children			
Age (m) ^a^	6.59 ± 5.15	6.45 ± 5.30	0.827
Gender			0.311
Male	76	39	
Female	141	56	
Parents			
Gender			0.805
Father	121	51	
Mother	96	44	
Marital status			0.304
Married	217	94	
Unmarried	0	1	
Divorced	0	0	
Mother’s education level			0.464
Lower education	34	19	
Intermediate education	111	42	
Higher education	72	34	
Father’s education level			0.423
Lower education	39	20	
Intermediate education	106	41	
Higher education	73	34	

*n* number of individuals, *m* month

^a^ Values are presented as mean (range)

**Table 2:** Clinical characteristics and demographic details of patients with infantile hemangiomas

Characteristics	*n*
Patients	
Child’s age (m)	
>0;≤6	132
>6;≤12	63
>12;≤18	13
>18;≤24	9
Infantile hemangiomas	
Location	
Extremities	36
Trunk	44
Diaper are	12
Craniofacial and neck	109
Internal organs	16
Number of lesions	
≥ 1;≤2	212
≥ 3;≤4	3
≥ 5	2
Size (cm^2^)	
>0;≤2	111
>2;≤4	30
>4;≤6	33
>6	43

*m* month

Scores of each instrument and each dimension are shown in Table 3. We found no significant difference in GCIS total scores between patient group and healthy group (*P* = 0.409). There were also no significant differences between two groups in scores of physical functioning (*P* = 0.872), emotional functioning (*P* = 0.734), social functioning (*P* = 0.846) and cognitive functioning (*P* = 0.835). However, there was significant difference in scores of physical symptom dimension between two groups (*P* = 0.021).

**Table 3:** Scores of each instrument in patient group and healthy group

Variables	Patient group (mean ± SD)	healthy group (mean ± SD)	*P*-Values
IH-QOL	84.79 ± 12.75	N/A	N/A
Physical symptom of patient	90.13 ± 14.72	N/A	N/A
Social functioning of patient	85.49 ± 17.60	N/A	N/A
Social and psychological functioning of caregiver	89.03 ± 15.50	N/A	N/A
Emotional functioning of caregiver	74.50 ± 18.55	N/A	N/A
PedsQL™ 4.0 Family Impact Module	83.25 ± 16.13	N/A	N/A
Physical functioning	82.66 ± 19.34	N/A	N/A
Emotional functioning	83.55 ± 21.02	N/A	N/A
Social functioning	85.54 ± 17.68	N/A	N/A
Cognitive functioning	82.12 ± 20.82	N/A	N/A
Communication	90.17 ± 15.11	N/A	N/A
Worry	73.55 ± 23.07	N/A	N/A
Daily activities	80.41 ± 22.63	N/A	N/A
Family relationships	88.80 ± 17.23	N/A	N/A
Financial issues	82.49 ± 23.67	N/A	N/A
PedsQL™ 4.0 Generic Core Infant Scales	88.62 ± 11.53	89.68 ± 6.16	0.409
Physical functioning	91.64 ± 12.43	91.41 ± 9.01	0.872
Physical symptom	86.48 ± 12.98	90.29 ± 10.78	0.021
Emotional functioning	84.29 ± 15.70	85.11 ± 15.44	0.734
Social functioning	92.14 ± 11.81	91.95 ± 12.03	0.846
Cognitive functioning	89.11 ± 17.38	89.63 ± 13.92	0.835

^*^ *SD* standard deviation, *N/A* not available

Table 4 demonstrates the internal reliability estimates of IH-QOL summary scale and subscales. The Cronbach’s alpha coefficient for IH-QOL summary scores was 0.76. The Cronbach’s alpha coefficient for IH-QOL subscale scores ranged from 0.65 to 0.75. Item-summary correlation ranged from 0.482 to 0.686, which meant strong correlation.

**Table 4:** Cronbach’s alpha coefficient for IH-QOL

Scale	Item-summary correlation	Cronbach’s alpha coefficient
Summary scale	N/A	0.76
Child physical symptoms	0.489	0.75
Child social interactions	0.482	0.75
Parent psychosocial functioning	0.686	0.65
Parent emotional functioning	0.622	0.68

*N/A* not available

IH-QOL total score was moderately related to GCIS total score with Pearson correlation coefficient being 0.582 (*P* < 0.05) (Table 5). In addition, IH-QOL total score was strongly related to FIM total score with Pearson correlation coefficient being 0.713 (*P* < 0.05) (Table 6).

**Table 5:** Pearson correlation coefficients between PedsQL™ Family Impact Module and IH-QOL ^a^

PedsQL™ Family Impact Module	IH-QOL				
	Physical symptom of patient	Social functioning of patient	Social and psychological functioning of caregiver	Emotional functioning of caregiver	Summary score
Physical functioning	0.452	0.309	0.584	0.553	0.616
Emotional functioning	0.460	0.347	0.621	0.644	0.675
Social functioning	0.401	0.352	0.565	0.484	0.585
Cognitive functioning	0.365	0.260	0.505	0.515	0.536
Communication	0.313	0.326	0.535	0.502	0.548
Worry	0.323	0.432	0.487	0.668	0.633
Daily activities	0.376	0.270	0.514	0.523	0.548
Family relationships	0.396	0.325	0.563	0.470	0.569
Financial issues	0.341	0.245	0.401	0.411	0.455
Summary score	0.377	0.335	0.634	0.667	0.713

^a^Values denote Pearson correlation coefficients. The *P* values of all coefficients above are less than 0.05

**Table 6:** Pearson correlation coefficients between PedsQL™ 4.0 Generic Core Infant Scales and IH-QOL ^a^

PedsQL™ 4.0 Generic Core Infant Scales	IH-QOL				
	Physical symptom of patient	Social functioning of patient	Social and psychological functioning of caregiver	Emotional functioning of caregiver	Summary score
Physical functioning	0.451	0.323	0.355	0.388	0.498
Physical symptom	0.426	0.265	0.359	0.525	0.523
Emotional functioning	0.466	0.249	0.486	0.505	0.560
Social functioning	0.261	0.234	0.323	0.304	0.371
Cognitive functioning	0.338	0.201	0.418	0.347	0.427
Summary score	0.471	0.308	0.479	0.504	0.582

^a^Values denote Pearson correlation coefficients. The *P* values of all coefficients above are less than 0.05

The outcomes of multiple step-wise regression analyses for IH-QOL total scores are listed in Table 7. We found the main influencing factors for IH-QOL total scores included hemangioma location, hemangioma size and mother’s education level. For GCIS total scores, the main influencing factors included child’s age, hemangioma location and father’s education level (Table 8). For FIM total scores, the main influencing factors included hemangioma location and father’s education level (Table 9).

**Table 7:** Factors influencing IH-QOL total score in patients with infantile hemangiomas ^a^

Factors	Score (mean ± SD)	β-Values (95%CI)	*P*-Values
Child’s gender		−0.024 (−4.111–2.8760)	0.728
Female	84.43 ± 12.84		
Male	85.47 ± 12.62		
Child’s age (month)		0.088 (−0.115–0.541)	0.202
> 0;≤6	83.89 ± 13.13		
> 6;≤12	86.33 ± 12.67		
> 12;≤18	85.84 ± 9.74		
> 18;≤24	85.63 ± 11.88		
Hemangioma location		0.148 (0.034–1.487)	0.04
Extremities	87.67 ± 9.57		
Trunk	87.73 ± 11.17		
Diaper area	85.46 ± 12.94		
Visceral involvement	83.12 ± 13.65		
Craniofacial and neck	81.10 ± 14.97		
Number of hemangioma		−0.091(−6.058–1.321)	0.207
≥ 1;≤2	85.00 ± 12.39		
≥ 3;≤4	81.24 ± 18.44		
≥ 5	68.28 ± 37.79		
Hemangioma size (cm^2^)		−0.167 (−0.563--0.062)	0.015
> 0;≤2	87.53 ± 10.81		
> 2;≤4	82.52 ± 13.42		
> 4;≤6	81.40 ± 13.18		
> 6	81.91 ± 15.24		
Mother’s education level		0.287 (0.495–5.989)	0.021
Lower education	82.39 ± 13.82		
Intermediate education	84.59 ± 13.48		
Higher education	86.25 ± 10.85		
Father’s education level		−0.217 (−4.974–0.203)	0.071
Lower education	83.21 ± 14.00		
Intermediate education	85.38 ± 13.33		
Higher education	84.73 ± 11.23		

^a^ Multiple step-wise regression analyses were used to find out the main risk factors

**Table 8:** Factors influencing PedsQL™ 4.0 Genetic Core Infant Scales total score in patients with infantile hemangiomas ^a^

Factors	Score (mean ± SD)	β-Values (95%CI)	*P*-Values
Child’s gender		0.027 (−2.956–4.263)	0.358
Female	88.88 ± 10.54		
Male	88.13 ± 13.35		
Child’s age (month)		−0.156 (−0.666--0.008)	0.045
> 0;≤6	89.72 ± 10.70		
> 6;≤12	87.86 ± 11.74		
> 12;≤18	83.67 ± 15.08		
> 18;≤24	85.36 ± 15.23		
Hemangioma location		0.179 (0.111–1.676)	0.025
Extremities	91.74 ± 9.36		
Trunk	88.72 ± 10.78		
Visceral involvement	88.42 ± 11.30		
Craniofacial and neck	87.02 ± 12.63		
Number of hemangioma		−0.127 (−6.506–0.684)	0.112
≥ 1;≤2	88.78 ± 11.41		
≥ 3;≤4	89.11 ± 18.00		
≥ 5	74.69 ± 9.63		
Hemangioma size (cm^2^)		0.062 (−0.154–0.373)	0.412
> 0;≤2	89.02 ± 11.58		
> 2;≤4	84.52 ± 13.00		
> 4;≤6	91.44 ± 11.16		
> 6	88.12 ± 10.03		
Mother’s education level		0.198 (−0.809–4.939)	0.158
Lower education	88.90 ± 11.67		
Intermediate education	88.32 ± 11.31		
Higher education	89.03 ± 12.06		
Father’s education level		−0.275 (−5.392--0.074)	0.044
Lower education	90.46 ± 10.72		
Intermediate education	88.07 ± 11.73		
Higher education	88.36 ± 11.80		

^a^ Multiple step-wise regression analyses were used to find out the main risk factors

**Table 9:** Factors influencing PedsQL Family Impact Module total score in patients with infantile hemangiomas ^a^

Factors	Score (mean ± SD)	β-Values (95%CI)	*P*-Values
Child’s gender		0.032 (−0.3445–5.582)	0.641
Female	83.42 ± 15.96		
Male	82.79 ± 16.48		
Child’s age (month)		−0.036 (−0.537–0.312)	0.602
> 0;≤6	83.52 ± 16.93		
> 6;≤12	83.28 ± 14.95		
> 12;≤18	82.73 ± 12.32		
> 18;≤24	79.64 ± 18.66		
Hemangioma location		0.155 (0.08–1.959)	0.033
Extremities	84.49 ± 20.13		
Trunk	83.88 ± 19.23		
Visceral involvement	83.74 ± 16.41		
Craniofacial and neck	81.82 ± 15.72		
Number of hemangioma		−0.121 (−8.842–0.693)	0.094
≥ 1;≤2	83.50 ± 16.00		
≥ 3;≤4	77.69 ± 18.48		
≥ 5	65.26 ± 27.33		
Hemangioma size (cm^2^)		−0.114 (−0.596–0.051)	0.098
> 0;≤2	86.42 ± 13.00		
> 2;≤4	79.11 ± 17.61		
> 4;≤6	84.19 ± 15.01		
> 6	77.25 ± 20.82		
Mother’s education level		0.227 (−0.251–6.849)	0.068
Lower education	83.34 ± 15.92		
Intermediate education	82.53 ± 16.93		
Higher education	84.28 ± 14.95		
Father’s education level		−0.249 (−6.850--0.160)	0.040
Lower education	85.19 ± 14.59		
Intermediate education	83.22 ± 15.98		
Higher education	82.32 ± 17.18		

^a^ Multiple step-wise regression analyses were used to find out the main risk factors

## Discussion

As experience treating IHs with β-blockers increases, a careful shift to more cosmetic indications is occurring. However, in spite of its efficacy, use of β-blockers has some risk [16–20]. This dilemma makes clinicians face new and difficult challenges when deciding whether or not to use β-blockers in small IHs when parents are worried about their child’s visible lesion, particularly present on the face. In this regard, QOL should be the important measurement to resolve the challenges and to evaluate the efficacy of treatment. Although several scoring systems for IH have been described over the years, there have been few studies describing QOL in IH patients and their parents [21, 22]. In addition, only a few common instruments, like the PedsQL™, could be used to assess the QOL in IH patients before IH-QOL developed. There is an emerging perspective that both generic and disease-specific instrument should be administered to IH patients and their families. For this reason, we chose to use both generic and disease-specific instrument in current study.

PedsQL™ is thought to be a reliable valid modular approach to evaluating QOL in children with or without disease [23, 24]. In the present study, we used the GCIS to evaluate the QOL in IH patients. Our results demonstrated that there was no significant difference in total score between IH patients and healthy children. These findings were inconsistent with report by Cohen-Barak et al. [7], who showed that IH patients and their parents even reported higher QOL than healthy children. In addition, we found physical symptom scores in patient group were significant lower than that of healthy children. One explanation for these obvious discrepancies is that Chinese parents seem to care more about the physical effects of IH on their children. Second, we have included patients with visceral IH. These patients can be associated with comorbid diseases and are more commonly to be managed aggressively with medical therapy [4, 25, 26]. Third, it might attribute to the marked age differences between two studies: in the present study, our children were too young to report on their own behalf. Only parent proxy-report was available. However, although children-self-reporting is important, perspective of parents also are critical. Furthermore, it is usually parents’ perceptions of children’s QOL that influences health care utilization [11, 27].

On the other hand, our data suggested that PedsQL™ GCIS might not be able to accurately evaluate QOL in IH patients alone. Hence, disease-specific instrument should be used to evaluate the QOL in IH patients as well. Our study showed Chinese version of IH-QOL can be completed smoothly with few questions by most of parents. The internal reliability for the IH-QOL summary and subscales scores was excellent, with a high degree of internal consistency among all item scores. In addition, our study found that IH-QOL was significantly correlated with GCIS and FIM. These findings are exciting, suggesting that these instruments can be used in combination. The data from these standardized instruments can provide more information than when they are used alone.

In keeping with the previous studies, our multiple step-wise regression analyses demonstrated that hemangioma location and size were related to QOL [6, 8]. Remarkably, hemangioma location was the only factor influencing the total scores of all three instruments. We found that patient with IH in craniofacial and neck area had lower IH-QOL score than those with IH in trunk, diaper area or extremities. This finding is not surprising given craniofacial and neck hemangiomas are visible and carry a high risk of residua scar than in other sites [28]. Many craniofacial and neck lesions can leave residual scars, which may have life-long effects [29]. In addition, we found that when hemangioma size was >2 cm^2^, the IH-QOL total score decreased significantly. Therefore, for craniofacial and neck hemangioms with size >2 cm^2^, early intervention may be justified to potentially arrest the growth of the lesion, and avoid psychosocial concerns [30]. The introduction of relatively safer topical agents (e.g., timolol) now allows earlier and easier intervention in appropriate cases [31–33].

It is noteworthy that parents with higher education level reported significantly higher QOL values. Currently, accurate information for IH is disseminated widely via the Internet. Conceptually, parents with higher education levels may spend more time learning hemangioma characteristics by themselves. This finding is interesting, indicating that parents who worry about the QOL of their child can be reassured. In contrast, parents with lower education levels may have poor knowledge of IH. This in turn, causes the heightened anxiety even for those IHs that are unlikely to cause complication or leave scarring. On the other hand, clinician may wish to counsel the parents regarding the changes of IH, such as rapid growth, infection or ulceration, and to establish with the parents a means to see the child on short notice if such changes are observed [30, 34]. Furthermore, parental attitudes and beliefs are critical factors in treatment selection, more so in elective cases [2]. Thus, parental education on IH will not only directly improve the QOL, but may also indirectly improve QOL by preventing functional and/or cosmetic complications via early referral and timely intervention during the ‘watchful waiting’ period.

## Conclusion

In conclusion, this study emphasizes that IH can influence the QOL in young patients and their parents. Our results demonstrate that PedsQL GCIS may not be sufficient to evaluate QOL in children with IH alone. IH-QOL, with excellent internal reliability, is significantly associated with the PedsQL GCIS and FIM. In addition, our study provides novel findings that children’s age, hemangioma location and size, and parents’ education level are important risk factors for poor QOL in patients and their parents.

## Abbreviations

FIF: The PedsQL™ family information form
FIM: The pedsQL™ 4.0 family impact module
GCIS: The PedsQL 4.0 genetic core scales
IH: Infantile hemangioma
IH-QOL: The quality-of-life instrument for IH
PedsQL™: The pediatric quality of life inventory™
QOL: Quality of life
